# *NOTCH2* gene mutation and gamma-secretase inhibitor in mediating the malignancy of ovarian cancer

**DOI:** 10.18632/aging.205045

**Published:** 2023-09-19

**Authors:** Wenjing Wang, Ruiqian Liu, Wei Liao, Landie Ji, Jie Mei, Dan Su

**Affiliations:** 1 School of Medicine, University of Electronic Science and Technology of China, Chengdu 610054, Sichuan, China; 2 Deyang People’s Hospital, Deyang 618099, Sichuan, China; 3 Department of Gynecology and Obstetrics, Sichuan Academy of Medical Sciences and Sichuan Provincial People’s Hospital, University of Electronic Science and Technology of China, Chengdu 610054, Sichuan, China; 4 Chinese Academy of Sciences Sichuan Translational Medicine Research Hospital, Chengdu 610054, Sichuan, China

**Keywords:** ovarian cancer, cell malignancy, whole-exome sequencing, NOTCH2, mutation

## Abstract

The carcinogenic mechanisms by which serous ovarian cancer (OC) occurs remain to be explored. Currently, we have conducted whole-exome sequencing (WES) and targeted deep sequencing to validate new molecular markers, including *NOTCH2*, that impede the progression of cell malignancy in ovarian cancer (OC). Following *NOTCH2* P2113S mutation and *NOTCH* signaling pathway inhibitor N-[N-(3,5-difluorophenacetyl)-L-alanyl]-S-phenylglycine t-butyl ester (DAPT) treatment, the cell proliferation, migration, and invasion of A2780 and SKOV3 OC cells were examined *in vitro*. WES identified the P2113S point mutation in *NOTCH2*. The *NOTCH2* mutation rate was 26.67 % among the 75 OC cases. The *NOTCH2* P2113S mutation and DAPT treatment downregulated Notch-2 protein levels in the two OC cells. Functionally, interfering with *NOTCH2* expression promoted the migrative, proliferative, and invasive capacities of OC cells. Western blotting further confirmed that *NOTCH2*-mediated tumorigenesis lies in reducing apoptosis through dysregulation of Bax/Bcl2, affecting repair of DNA damage through reducing DNA-PK and blocking the transcription factor Hes1 along with increasing immune regulator p65. Furthermore, the *NOTCH2*-mediated tumorigenesis was mostly reversed after NF-κB inhibitor Bay11-7082 treatment. These findings identified the *NOTCH2* P2113S mutation in ovarian carcinogenesis, and *NOTCH2* P2113S is a potential target in treating OC.

## INTRODUCTION

Ovarian cancer (OC), the deadliest gynecologic malignancy [1], inflicts approximately 225,500 new cases and causes 140,200 associated deaths this year, as reported by the World Health Organization (WHO) [2]. Commonly found in stage III or IV women, high-grade serous OC (HGSOC) has a five-year survival ranging from 26 to 42% [3]. The exploration of various targets has enabled the development of new therapies such as targeted therapy. Compared with noncarriers, HGSOC patients with BRCA1 and BRCA2 mutations have benefited enormously from olaparib therapy since these mutations were identified [4]. As clinical genetics develops, novel candidate genes are urgently needed to improve OC management.

Using multigene panels for gene mutation assessment, many novel and significant variants have been discovered recently. Although exons make up only a small percentage of all genomic DNA (1.5%), they are involved in the majority of human diseases [5]. The current research utilized whole-exome sequencing (WES) and targeted deep sequencing (TDS) to identify candidate genes for rare mutations associated with serous OC (SOC). WES is an effective diagnostic method for individuals lacking classical pathogenic molecular changes, especially for diseases with high genetic heterogeneity, such as breast cancer and ovarian cancer susceptibility syndrome [6]. Furthermore, these mutations’ functions in oncogenesis and their influences on this process were determined by *in vitro* assays.

*NOTCH* signaling contributes not only to cell line age specification, tissue patterning and morphogenesis but also to stem cell maintenance and tissue homeostasis during embryonic development or in adult life [7]. In vertebrates, the *NOTCH* system comprises four receptors (Notch1-4) and at least five ligands (Delta-like [Dll]-1, Dll-3, Dll-4, Jagged-1 and Jagged-2) from the Delta and JAG/Serrate (DSL) families [8]. *NOTCH* signaling acts on cardiovascular and endocrine functions and the nervous system, directly influencing age-related diseases [9]. New treatment strategies have also been proposed to inhibit *NOTCH* signaling to prevent cancer recurrence and promote a cure [8]. Recent evidence has shown that the combination of the *NOTCH* inhibitor DAPT (N-[N-(3,5-Difluorophenacetyl)-L-alanyl]-S-phenylglycinet-butylester), a γ-secretase inhibitor, with other anti-inflammatory drugs and steroids can promote treatment efficiency [10]. It was also reported that *NOTCH2* was associated with OC [11] and female genital system diseases [12], indicating that *NOTCH2* plays a role in OC development.

Pharmacological inhibition of *NOTCH* signaling applying the γ-secretase inhibitors has shown clinical significance in treating tumors, and the potentials of DAPT-mediated antitumor activity have also been identified. Here, the motivation and novelty of the present study is to examine the therapeutic usage of DAPT combined with *NOTCH2* mutation in serous OC through WES, TDS and *in vitro* experiments. We also assumed that both *NOTCH2* gene mutation and DAPT alter the malignant behaviors of OC cells, and DAPT can further affect the malignance of OC cells with *NOTCH2* gene mutation.

## RESULTS

### WES determination of mutated genes in OC specimens

**Figure 1:**
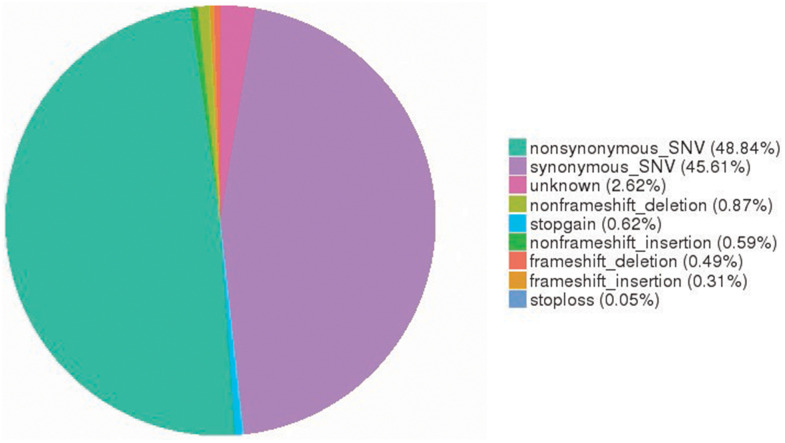
**Somatic SNV signatures in OC.** A total of 52,464 somatic SNVs (25,623 nonsynonymous and 23,929 synonymous SNVs) were identified in OC exomes. SNV, single nucleotide variant; OC, ovarian cancer.

To profile somatic mutations, we performed WES to examine 11 paired SOC tissue specimens and adjacent counterparts. In OC exomes, we identified 52,464 somatic single nucleotide variants (SNVs) (25,623 nonsynonymous and 23,929 synonymous SNVs). Of them, nonframeshift deletions, stop-gain mutations, nonframeshift insertions, frameshift deletions, frameshift insertions, and stop-loss mutations were found in 456, 325, 310, 257, 163, and 26, respectively. Among SOC substitution types, the C>T substitution was found to have the highest frequency. Furthermore, TCN site C>T mutations, especially in TCA-containing sequences, predominated in trinucleotide signatures (Figure 1).

**Figure 2:**
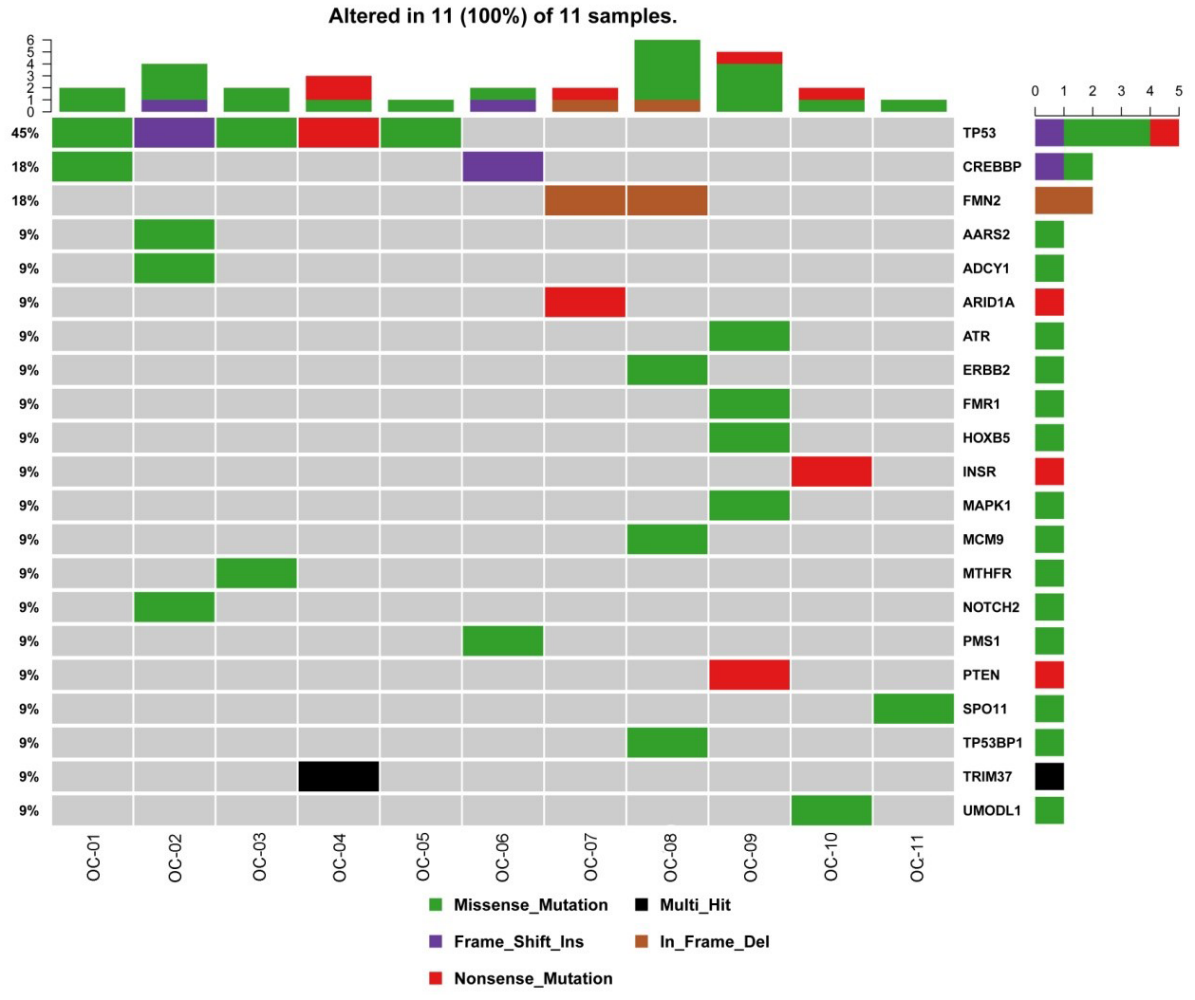
**Gene mutations in OC.** The top bar plot and the bottom left plot represent average somatic mutations and somatic mutation frequency, respectively. In the bottom middle plot, rows and columns denote individual genes and tumors, respectively. In the bottom right plot, mutations are colored by mutation type. OC, ovarian cancer.

In OC samples, 21 nonsilent mutations with higher detection rates were identified, including *TP53* (45%), *CREBBP* (18%), *FMN2* (18%) and *NOTCH2* (9%) (Figure 2). Since there are previous reports on *TP53* mutations (reported in more than 50% of human tumors [13]) and the *CREBBP and FMN2* genes, they were not selected as candidates. *NOTCH2* is associated with cancers and female genital system diseases according to the databases [12]; therefore, the *NOTCH2* gene was selected for the following experiments. One point mutation in the *NOTCH2* gene was identified in 1 case, p. P2113S (120459008: G>A), which is a missense mutation and nonsynonymous SNV. P2113Sis a new discovery that has no previous reports.

### Detection of *NOTCH2* mutations in clinical specimens by next-generation sequencing (NGS)

*NOTCH2* sequencing in 220 samples (64 III–IV SOC cases confirmed by postoperative pathology and 156 blood specimens from healthy control women) was performed using NGS. Combining WES data and TDS data, 75 specimens were evaluated (11 samples in WES and 64 samples in TDS); *NOTCH2* mutations were determined in 20 samples (1 in the WES analysis and 19 in the TDS analysis) with a rate of 26.67 % (20 of 75) among cancer cases. The *NOTCH2* mutation rate in blood samples of 156 healthy controls (41.03%) was significantly higher than that in OC cases (χ^2^ = 4.513, *P*= 0.034).

### The *NOTCH2* P2113S mutant inhibited Notch2 expression

**Figure 3:**
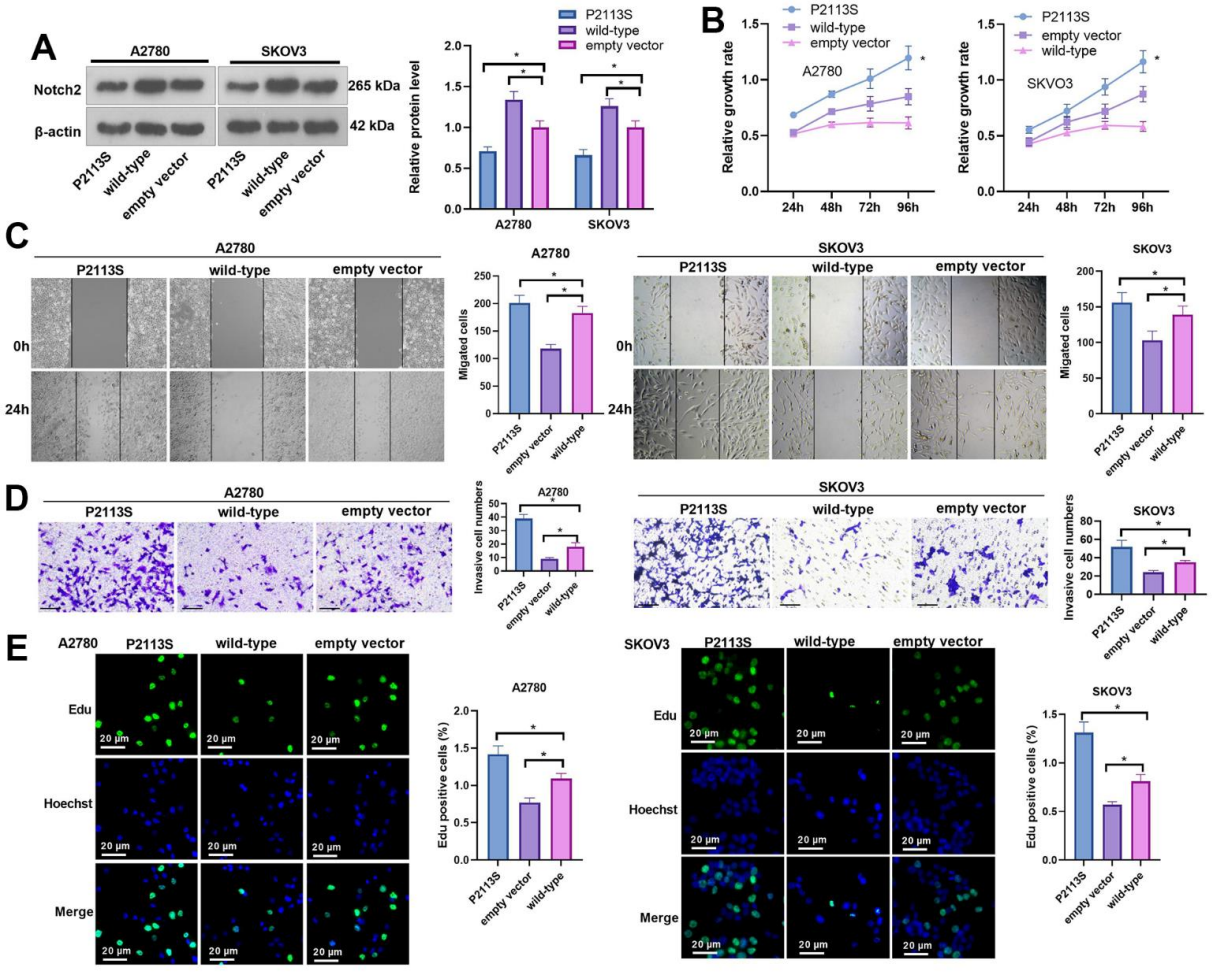
**Impacts of *NOTCH2* P2113S on A2780 and SKOV3 cell proliferation, migration, and invasion.** (**A**) Notch2 level was tested following transfection with *NOTCH2-*P2113S in A2780 and SKOV3 cells; (**B**) CCK-8 assay was used for detecting cell proliferation (*p<0.05 vs empty vector); (**C**) Scratch wound-healing assay showed alterations of cell migration. Upper panel, representative images (×100); Lower panel, quantitative analysis (*p<0.05); (**D**) Transwell chamber assay was used for evaluating cell invasion by *NOTCH2* P2113S transfection. Upper panel, representative images (×100); lower panel, quantitative analysis (*p<0.05). (**E**) EdU labeling test was used for testing cell proliferation. N=3.

To verify the tumorigenicity of the *NOTCH2* mutation, human SOC A2780 and SKOV3 cells were transfected with P2113S (120459008: G>A) mutant-carrying plasmids. WB analysis revealed increased Notch2 protein in A2780 and SKOV3 cells by WT *NOTCH2* transfection compared with empty vector transfection. P2113S mutant overexpression caused a decrease in Notch2 protein expression compared with the empty vector (Figure 3A, *P*< 0.05, β-actin molecular weight: 42 kDa and Notch-2 molecular weight: 265 kDa). These results demonstrated that the *NOTCH2* P2113S mutant inhibited Notch2 protein expression in A2780 and SKOV3 cells.

### The *NOTCH2* P2113S mutant promoted A2780 and SKOV3 cell proliferation, migration, and invasion

To detect the influence of *NOTCH2* mutation on OC cell proliferation, the relative cell growth rate of each group was detected by CCK8 assay at 24, 48, 72 and 96 h post transfection. As time went by, the cell growth rate in each group increased in a time-dependent manner (Figure 3B). When A2780 cells were transfected for 96 h, the proliferation activities of the WT group, empty vector group and P2113S group were 0.614±0.023, 0.849±0.074 and 1.196±0.107, respectively, and the difference was statistically significant (*p*<0.05). In SKOV3 cells, the proliferation activities of the WT group, empty vector group and P2113S group were 0.584±0.043, 0.874±0.070, and 1.164±0.010, respectively, with statistical significance (*p*<0.05; Figure 3B). Moreover, scratch wound-healing assay and Transwell assay showed that at 24 hours after transfection, the scratch cell count in the WT group was markedly decreased compared with that in the empty vector group (*p*<0.05; Figure 3C), and the scratch cell count in the P2113S mutant group was significantly increased (*p*<0.05; Figure 3C). Consistent results were also found in transwell assay (Figure 3D). EdU labeling showed that downregulated expression of *NOTCH2* was accompanied by an increased EdU-positive cell count (Figure 3E) when compared to the control groups (In A2780 cells: (1.42±0.11) % in P2113S group; (0.77±0.06) % in WT group; (1.09±0.07) % in empty vector group. In SKOV3 cells: (1.31±0.12) % in P2113S group; (0.57±0.03) % in WT group; (0.81±0.06) % in empty vector group;). These findings demonstrated that *NOTCH2* mutation could promote A2780 and SKOV3 proliferation and suggested that the *NOTCH2* gene might inhibit OC cell growth.

### DAPT combined with *NOTCH2* mutation reduces the expression of Notch2 protein

**Figure 4:**
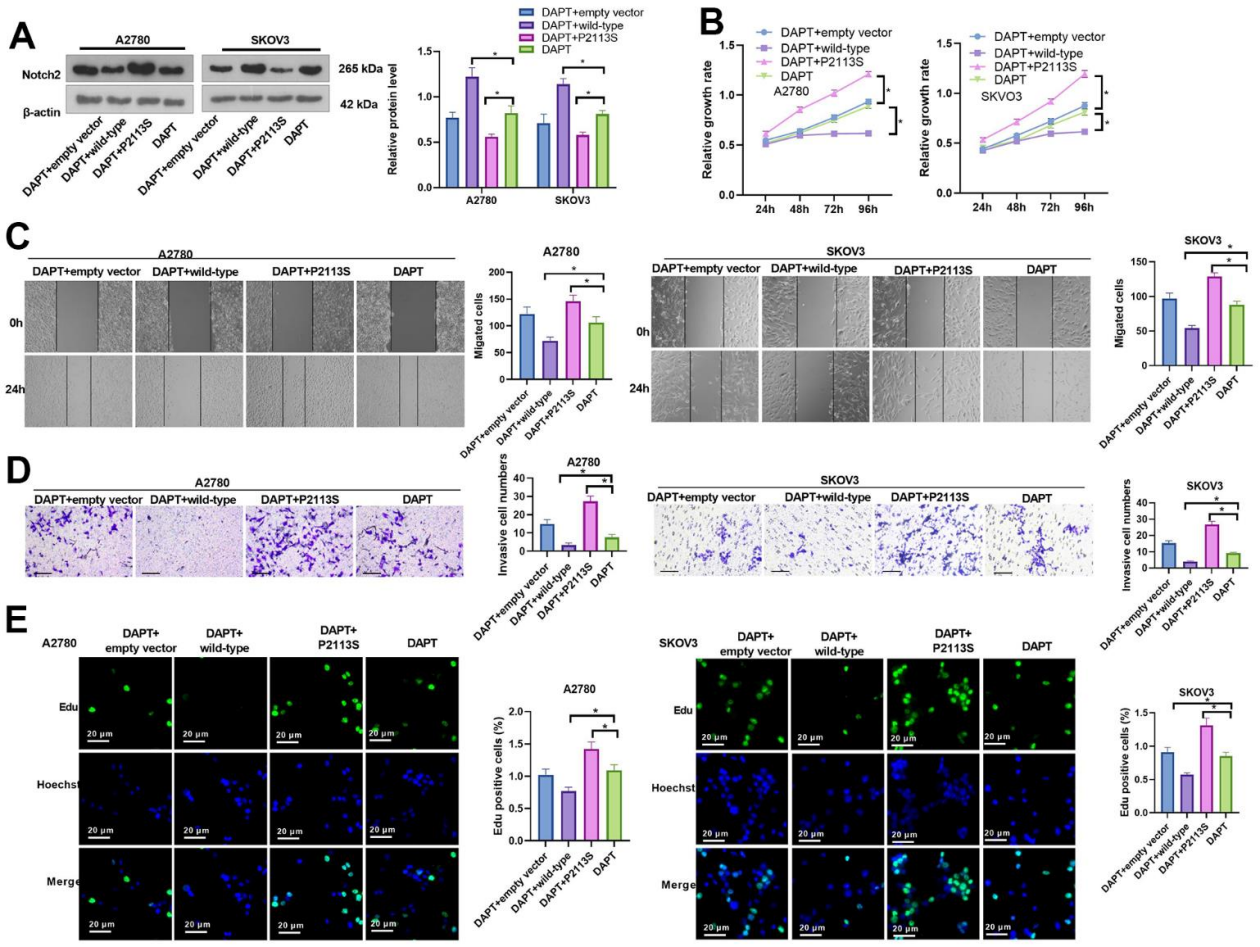
**Impacts of DAPT on *NOTCH2* mutated OC cells.** (**A**) Notch2 level was tested following transfection with *NOTCH2-*P2113S in A2780 and SKOV3 cells; (**B**) CCK-8 assay was used for detecting cell proliferation (*p<0.05); (**C**) Scratch wound-healing assay showed alterations of cell migration. Upper panel, representative images (×100); Lower panel, quantitative analysis (*p<0.05); (**D**) Transwell chamber assay was used for evaluating cell invasion by *NOTCH2* P2113S transfection. Upper panel, representative images (×100); lower panel, quantitative analysis (*p<0.05). (**E**) EdU labeling test was used for testing cell proliferation. N=3.

WB was utilized to identify Notch2 protein expression using the gamma-secretase inhibitor DAPT combined with the *NOTCH2* P2113S mutant. When DAPT combined with WT plasmids (DAPT+WT group) acted on A2780 and SKOV3 cells, the expression of Notch2 protein was higher than that in the DAPT + empty vector group (relative protein level in A2780 and SKOV3, respectively: 1.22±0.10 vs 0.77±0.06 and 1.14±0.06 vs 0.71±0.10); the expression of Notch-2 protein in the DAPT+P2113S group [relative protein level (0.56±0.03) in A2780 and (0.58±0.03) SKOV3] was significantly lower versus DAPT+ empty vector group (*p*<0.05; Figure 4A). There was no distinctive differentiation between the DAPT+ empty vector group and DAPT group [relative protein level (0.82±0.08) in A2780 and (0.81±0.04) SKOV3]. The results showed that the inhibitory effect of Notch2 expression used by combining *NOTCH2* P2113S and DAPT was enhanced.

### DAPT combined with *NOTCH2* mutation promoted OC cell proliferation, migration and invasion

The carcinogenesis ability of DAPT combined with *NOTCH2* P2113S (DAPT+P2113S group) was measured with CCK8, EdU, wound healing and transwell chamber assays between si-*NOTCH2* P2113S transfected and control groups, such as DAPT+empty vector group, DAPT+WT group and DAPT group. DAPT+P2113S significantly enhanced OC cell growth (Figure 4B), accelerated wound healing (Figure 4C), promoted invasion (Figure 4D) and increased the EdU-positive cell count (Figure 4E) compared to the control group. The above results indicated that DAPT combined with *NOTCH2* mutation promoted OC cell proliferation, migration and invasion *in vitro*.

### Changes in the expression of related proteins in A2780 and SKOV3 cells

**Figure 5:**
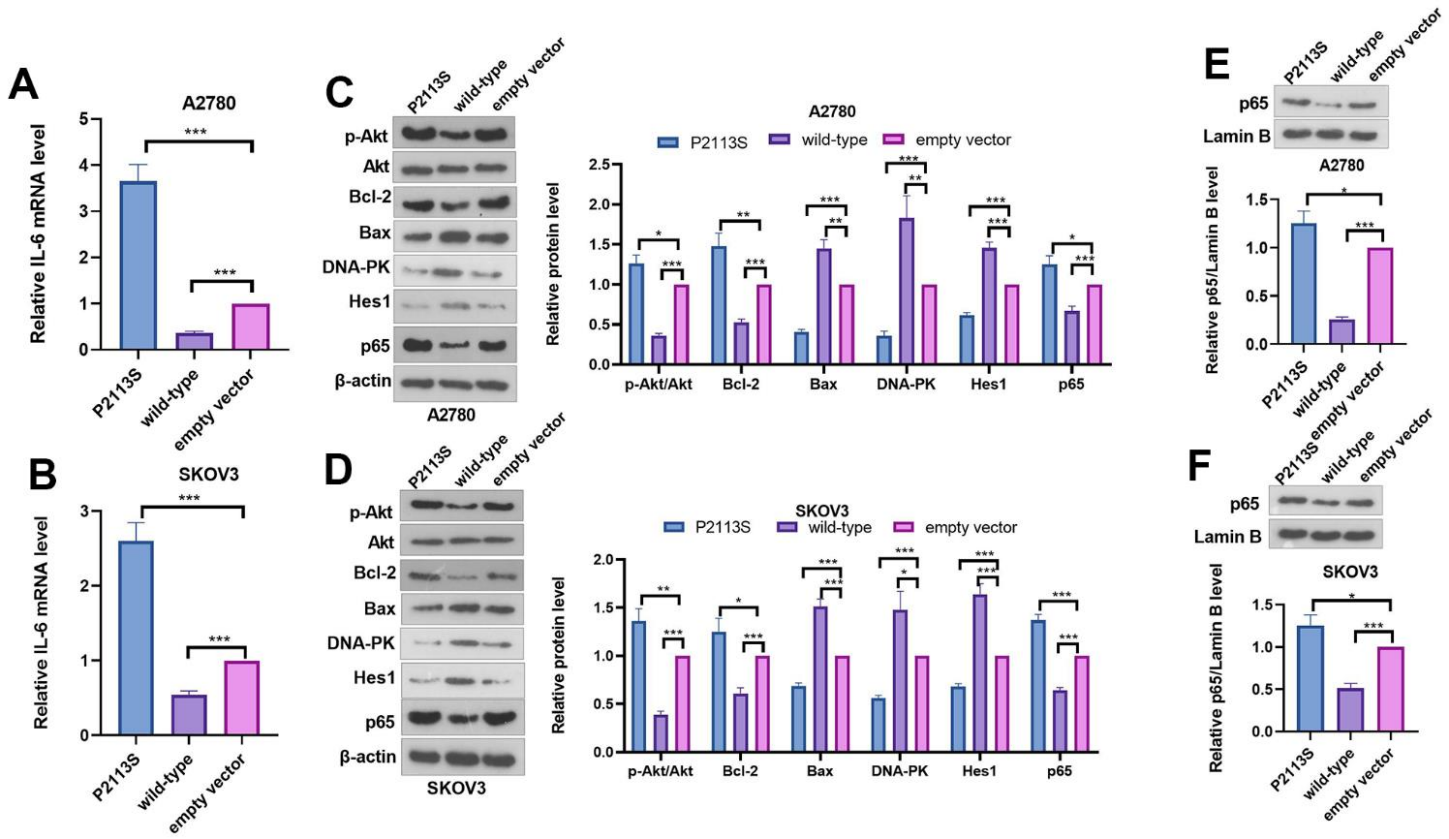
**Expression of related proteins in A2780 and SKOV3 cells.** (**A**, **B**) RT-PCR was used for evaluating IL-6 mRNA level. (**C**, **D**) WB analysis of p-Akt, Akt, Bcl-2, Bax, DNA-PK, Hes 1 and P65 proteins in OC cells. Each numerical value was the relative expression normalized to β-actin protein. (**E**, **F**) WB analysis of P65 proteins in the nucleus of OC cells. Each numerical value was the relative expression normalized to Lamin B protein. *p <0.05, **p <0.01, ***p <0.001. N=3.

WB was adapted to further investigate the expression of proteins related to tumorigenesis to examine the possible mechanism. IL-6 mRNA level was determined by qRT-PCR. The data indicated that P2113S treatment enhanced IL-6 mRNA level, which was reduced in the WT group (compared with the empty vector group, Figure 5A, 5B). The results of western blot showed obviously increased p-Akt, Bcl-2 along with significantly decreased Bax in cells subjected to P2113S treatment than in empty vector (Figure 5C, 5D). However, in the WT group, the opposite results were found, namely, p-Akt and Bcl-2 levels were decreased and Bax was increased. Next, DNA repair proteins, DNA-dependent protein kinase (DNA-PK) were examined by WB in A2780 and SKOV3 OC cells. DNA-PK was markedly decreased in the P2113S-transfected groups compared to the empty vector, while it was increased in the WT group (Figure 5C, 5D). Moreover, WB analysis found that P2113S transfection markedly reduced Hes1 protein while promoted p65 level when compared to empty vector transfection. WT transfection significantly promoted the expression of Hes1 protein when compared to controls (Figure 5C–5F). These results suggested that downregulated *NOTCH2* by P2113S might affect transcription by depressing Hes1 and enhancing p65.

### Inhibiting NF-κB pathway reversed *NOTCH2* mutation-promoted OC cell proliferation, migration and invasion

**Figure 6:**
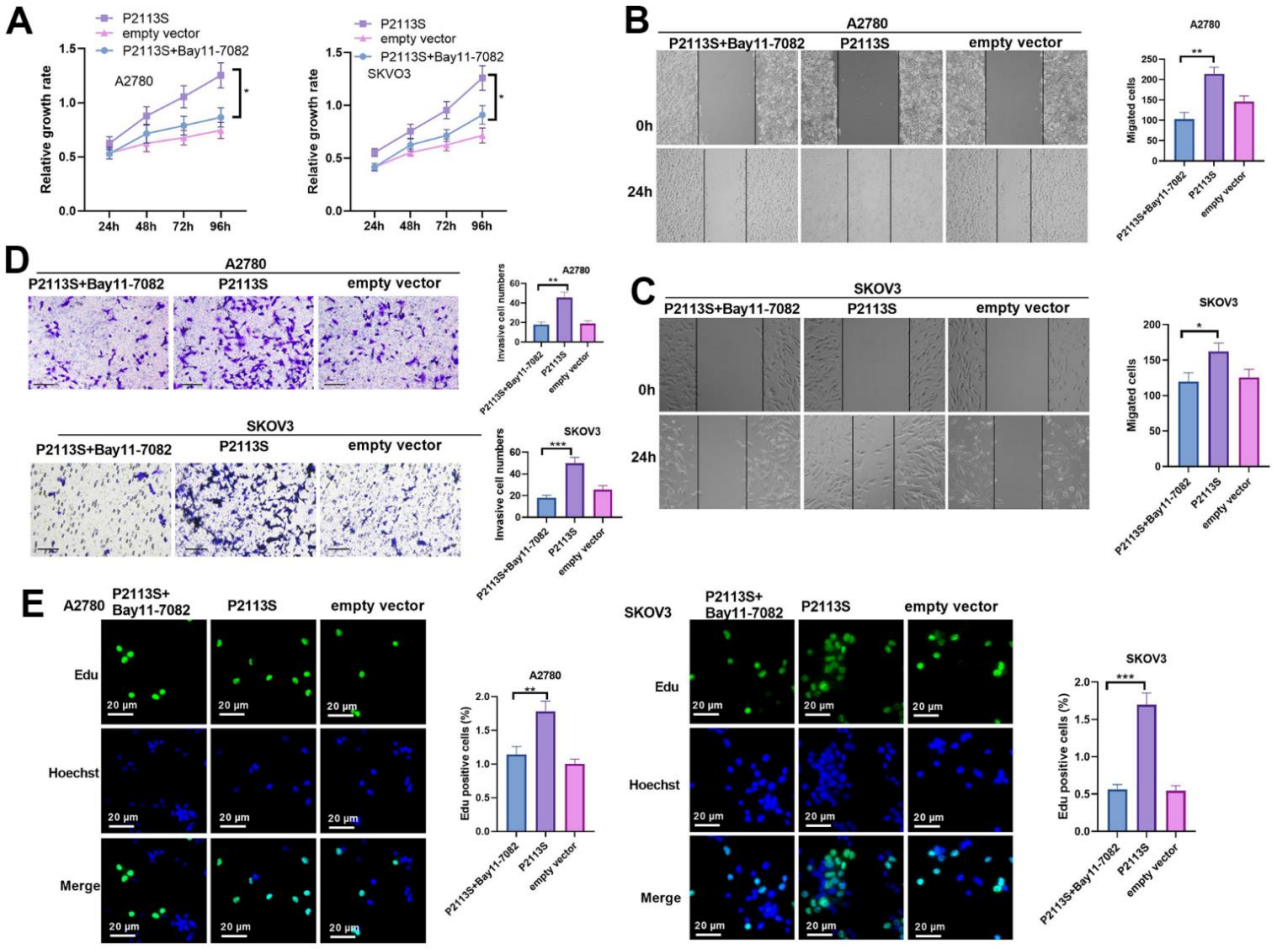
**Impacts of NF-κB inhibitor Bay 11-7082 on *NOTCH2* mutated OC cells.** (**A**) CCK-8 assay was used for detecting cell proliferation (*p<0.05); (**B**, **C**) Scratch wound-healing assay showed alterations of cell migration. Upper panel, representative images (×100); Lower panel, quantitative analysis (*p<0.05); (**D**) Transwell chamber assay was used for evaluating cell invasion by *NOTCH2* P2113S transfection. Upper panel, representative images (×100); lower panel, quantitative analysis (*p<0.05). (**C**) EdU labeling test was used for testing cell proliferation. N=3.

**Figure 7:**
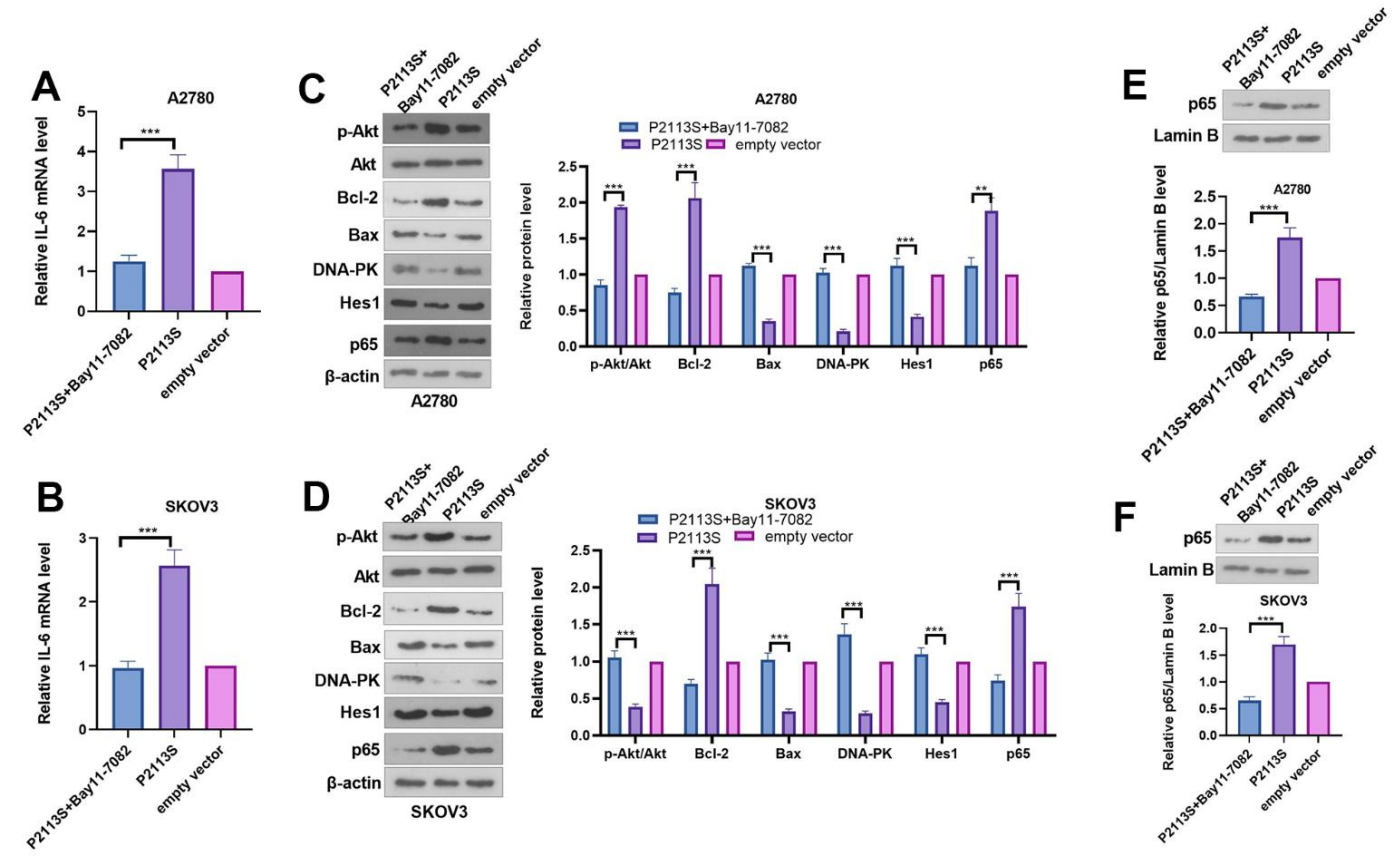
**Expression of related proteins in A2780 and SKOV3 cells.** (**A**, **B**) RT-PCR was used for evaluating IL-6 mRNA level. (**C**, **D**) WB analysis of p-Akt, Akt, Bcl-2, Bax, DNA-PK, Hes 1 and P65 proteins in OC cells. Each numerical value was the relative expression normalized to β-actin protein. (**E**, **F**) WB analysis of P65 proteins in the nucleus of OC cells. Each numerical value was the relative expression normalized to Lamin B protein. *p <0.05, **p <0.01, ***p <0.001. N=3.

To confirm the role of NF-κB in the carcinogenesis ability of *NOTCH2* P2113S, the NF-κB pathway inhibitor Bay11-0782 was administered into OC cells with *NOTCH2* mutation. As shown by the functional assay, Bay11-7082 addition reduced cell proliferation, migration, invasion (compared with the P2113S group, Figure 6A–6E). In addition, we tested the alterations of IL-6, Akt, Bcl-2, Bax, DNA-PK, Hes1, and p65 levels. The results showed that Bay11-7082 repressed IL-6, p-Akt, Bcl-2 and p65 levels, and promoted the profiles of Bax, DNA-PK and Hes1 (Figure 7A–7F). These results suggested that downregulated *NOTCH2* by P2113S depends on enhancing p65.

## DISCUSSION

**Figure 8:**
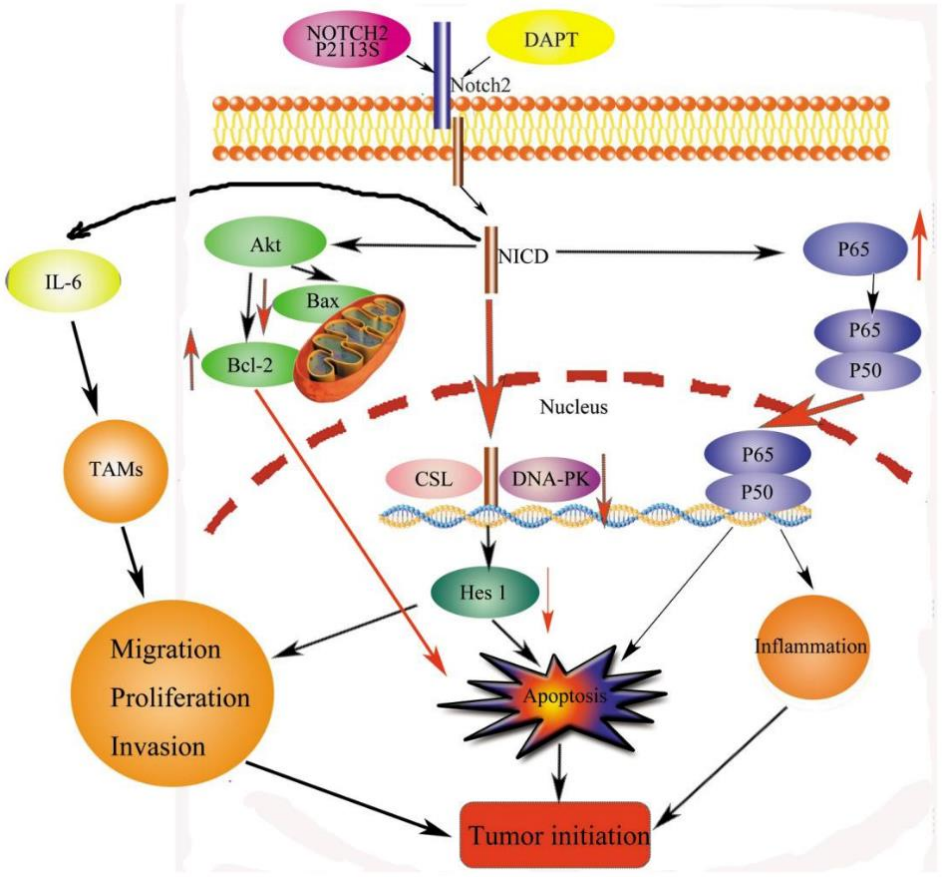
**Signaling pathways for tumorigenesis effects by *NOTCH2* mutation.** Apoptosis effects through the pAKT-Bax/Bcl-2 and pNF-κB/Rel pathways. Meanwhile, *NOTCH2* mutation promoted tumor cell migration, invasion, and proliferation through Hes 1 and TAMs involved in the tumor microenvironment (TME). Abbreviations: NICD: Notch2 intracellular domain; TAMs: tumor-associated macrophages.

In HGSOC patients, the five-year OS was approximately 45%. Different prognoses are linked to disease staging. For instance, the 5-year OS exceeds 70% in stage I and II HGSOC patients versus 26–42% in advanced stage (III and IV) individuals [3]. New treatments such as precision oncology therapy have been developed. Clinical applications of precision oncology require accurate tests that can distinguish true cancer-specific mutations. WES is used to detect mutations relevant to cancer and find target genes [14]. We identified one point mutation in the *NOTCH2* gene in OC tissues by combining WES with NGS. Vectors carrying *NOTCH2* WT or the *NOTCH2* mutant P2113S were transfected into OC A2780 and SKOV3 cells. In addition to inhibiting Notch2 protein expression, the P2113S mutant promoted OC cell migration, invasion, and growth. To examine the applications of Notch2 inhibition on ovarian carcinogenesis, DAPT, a potent pharmacologic Notch inhibitor, was adapted on A2780 cells and SKOV3 cells. The results showed that DAPT combined with *NOTCH2* mutation enhanced the progression of carcinogenesis by promoting OC cells’ capacity to proliferate, migrate and invade. The possible mechanism by which downregulating *NOTCH2* leads to tumorigenesis might lie in the depression of Bax, DNA-PK and Hes 1 and the increased expression of Akt, Bcl-2 and P65 (Figure 8).

The *NOTCH* axis, an evolutionarily conserved intercellular communication mechanism, is mediated by the interplay between receptors and cognate ligands on the cell membrane of neighboring cells. The mutation rate of Notch 2 varies in different cells. According to George J et al. [15], inactivating mutations in *NOTCH* family genes were found in 25% of human small cell lung cancers (SCLCs). Consistently, this study identified *NOTCH2* mutations in 26.67% (20 of 75) of the cancer cases. Some mutant spots were detected. In desmoid tumors, the presence of CTNNB1 missense mutations of *NOTCH2* was found [16]. In another study, heterozygous mutations in *NOTCH2*, such as the nonsense mutation c.7198C>T in HCS06 and mutation c.6383delG in HCS02, were identified as the cause of Hajdu–Cheney syndrome, which affects several organ systems, leading to severe osteoporosis and other abnormalities [17]. Lin Li et al. [12] reported a rarely seen *NOTCH2* gene heterozygous variant (c.5557G>C;p. D1853H) as a pathogenic allele of premature ovarian insufficiency (POI). In this experiment, P2113S (120459008: G>A) was found to be related to OC.

In the present study, the *NOTCH2* P2113S mutation triggered malignancy by promoting cancer cells’ ability to migrate, invade, and proliferate. The involvement of the *NOTCH* axis in various cellular processes (cell differentiation, growth, survival, apoptosis [11] and stem cell maintenance [18]) has been well documented, with *NOTCH* family members acting as both oncogenes and antioncogenes. Furthermore, different Notch receptors can present opposite expression patterns and exert opposite effects in a single tumor type. Unlike *NOTCH1* and *NOTCH3*, *NOTCH2* has always been shown to play a tumor inhibitory role in multiple cancers, with its expression maintained at high levels in well-differentiated tumors and at low levels in poorly differentiated breast tumors, while silencing Notch2 reversed breast cancer cell proliferation [18]. In addition, *NOTCH2* exerts proapoptotic and growth-inhibiting effects in thyroid and carcinoid cancers. A marked antitumor effect of *NOTCH2* in MDA-MB-231 cells has also been proposed by O’Neill et al. [19]. However, in some other cancers, [15, 20] such as SCLC and embryonal brain tumors, *NOTCH2* may act as a tumor promotor. Stably downregulating *NOTCH2* in hepatocellular carcinoma cell lines leads to attenuated cell invasion and migration potential and tumorigenicity *in vivo,* accompanied by histological maturation [20]. In OC, Notch2 expression was decreased in cancerous tissues compared with adjacent counterparts and normal control tissues [21]. In the present study, *NOTCH2* mutation downregulated the expression of Notch2 in OC cells, resulting in promoted cell migration, invasion, and proliferation. This result indicated that *NOTCH2* was the tumor suppressor gene of OC, which agrees with past literature.

In this study, DAPT combined with *NOTCH2* mutation enhanced the progression of carcinogenesis by promoting OC cell migration, invasion, and proliferation. Gamma-secretase inhibitors (GSIs) can inhibit Notch signaling to target cancer-initiating genes and sensitize cancer cells to chemotherapy [22]. Hans CP et al. [23] reported that DAPT reverted proinflammatory genes back to baseline in macrophages and increased anti-inflammatory genes, including c-Myc, Egr2, and Arg1, in LPS-stimulated macrophages, preventing the progression of active abdominal aortic aneurysm (AAA). Dai G et al. [22] treated CDDP-resistant osteosarcoma cell lines with DAPT+CDDP and detected that DAPT enhanced the cytotoxic effect of CDDP in resistant osteosarcoma by suppressing proliferation, leading to G0/G1 cell cycle arrest, thus inducing apoptosis and inhibiting motility. Based on previous evidence, researchers have designed γ-secretase inhibitors, siRNAs, monoclonal Abs against the Notch pathway and other small molecule inhibitors for desmoid tumor therapy. In a phase II trial, favorable efficacy of PF-03084014 (a γ-secretase inhibitor) was demonstrated, with a response rate of 29% for progressive desmoid tumor patients [24]. Kang H, et al. [25] used siRNA knockdown and gamma-secretase inhibitor (GSI) on paclitaxel-resistant SKpac and parental SKOV3 cells and detected that Notch inhibition significantly boosted sensitivity to paclitaxel.

B-cell lymphoma-2 (BCL-2) family proteins are responsible for the regulation of the intrinsic apoptotic pathway. BAX and BAK, proapoptotic BCL-2 proteins, can induce programmed cell death and initiate caspase cascades. Recent intensive studies of BAX/BAK shuttling, BCL-2 protein interactions and active BAX complexes have laid the foundation for developing novel strategies for cancer therapy and analyzing cellular susceptibility to apoptosis [26]. Dysregulation of the BAX/BCL2 balance could induce apoptosis by activating proapoptotic BAX gene expression levels 10 times higher than those of BCL2 in OC [27]. Notch2-deficient human pulmonary artery endothelial cells activated Akt, Erk1/2 and the anti-apoptotic protein Bcl-2 and reduced the levels of p21cip and Bax associated with increased endothelial cell proliferation and reduced apoptosis [28]. Kang H, et al. [25] reported reduced anti-apoptotic protein (BCL-XL, BCL-W, and BCL2) expression and increased pro-apoptotic protein (Bad, Bid, Bak, Bax, and Bim) levels by Notch3 siRNA treatment. In this study, Bax was reduced and Bcl-2 was increased, indicating that *NOTCH2* was related to anti-apoptotic usage in OC.

DNA-PK interferes with multiple cellular pathways, playing a key role in cellular responses to DNA injury and repair of DNA double-strand breaks and consequently playing a vital role in genomic integrity maintenance. In addition, it can modulate transcription and participate in immune system development and telomere protection. This pleotropic involvement and its deregulated expression in cancers have enabled DNA-PK to be a target of interest in cancer therapy [29]. Research has linked decreased DNA-PK activity with cancer initiation due to defects in repair. However, higher DNA-PK levels and viability have also been found in various other cancer cells and have been linked to the decreased efficiency of antitumor drugs [29]. In this study, the expression of DNA-PK was reduced after transfection with P2113S and treatment with DAPT, suggesting that downregulating *NOTCH2* initiated OC through depression of DNA-PK.

Hes1, a transcription factor under the regulation of the *NOTCH*, Hedgehog and Wnt axes [30], belongs to the extensive family of basic helix-loop-helix (bHLH) proteins, which are essential in modulating the cell cycle, growth, differentiation, survival and apoptosis in endocrine, neuronal, and T-lymphocyte progenitors as well as various cancers. Downregulation of Notch/Hes1 signaling was associated with Notch-regulated dendritic cell immune responses (IRs) in a mouse colitis colorectal cancer model. Hes1 expression depletion is often observed in sessile serrated adenomas/polyps (SSA/p) and colorectal cancer [31]. *NOTCH1*, *NOTCH2* and *NOTCH4* were significantly downregulated in bladder cancer samples compared with controls. In contrast, *NOTCH3* and *HES1* were significantly overexpressed [16]. *NOTCH2* expression was strongly linked to *HES1* expression, while other *NOTCH* gene levels were not, as indicated by RNA-sequencing analysis of desmoid tumor samples. *NOTCH2* activation causes *HES1* overexpression, proliferation, immature morphology and invasion in an acute kidney injury model [32] and hepatocellular carcinoma model [20]. In this research, the expression of Hes1 was reduced after transfection with P2113S and treatment with DAPT, suggesting that downregulating *NOTCH2* initiated OC through depression of Hes1, consistent with the results but controversial with others. More studies should be conducted to clarify the relationship between *NOTCH2* and Hes1.

P65 (RelA), a member of the NF-κB/Rel family, is also a critical gene regulating immune and inflammatory responses. They form homo or heterodimers and maintain inactive complexes with inhibitory molecules called IκB proteins in resting cells. The p65:p50 heterodimer is the most abundant form of NF-κB activated by pathologic stimuli via the canonical pathway. Hence, the NF-κB p65 axis has been pivotal for intense drug discovery and development [33]. Many chronic inflammations, both infectious and noninfectious (or idiopathic), can lead to tumors. Cao Q, et al. [34] reported that *NOTCH1* might transactivate NF-κB/p65 by stimulating p65-dependent proinflammatory functions in suppressing microglia in rats. Notch-1, p-Akt and p-NF-κB p65 protein levels were downregulated in cisplatin-resistant OC cells. Zou W, et al. [35] showed significantly upregulated Notch-1 and NF-κB p65 protein levels in cisplatin-resistant OC cells through WB analysis, suggesting the involvement of Notch-1/Akt/NF-κB in OC chemoresistance. Lin L, et al. [36] reported that downregulation of p65 in hepatocellular carcinoma inhibits inflammatory responses and hepatocarcinogenesis. In this experiment, downregulating *NOTCH2* expression with P2113S increased p65 protein stability and enhanced NF-κB activation, promoting inflammatory cytokine release. We administered OC cells with NF-κB, and the functional and mechanistic studies suggested that inhibiting NF-κB reversed the oncogenic effects of *NOTCH2* P2113S mutation. This result suggested that Notch-2 signaling might transactivate NF-κB/p65 by stimulating p65-dependent proinflammatory functions in promoting OC growth.

Composed of immune cells and immune regulatory molecules, the tumor microenvironment (TME) is implicated in host antitumor IRs and immune suppressive mechanisms that promote cancer progression. In addition, Notch activity can disrupt neuroendocrine gene expression in SCLC cells. This is the first comprehensive study on somatic genome alterations in SCLC, revealing multiple critical biological processes and identifying candidate targets for this highly lethal cancer that are related not only to cellular intrinsic mechanisms but also to TME generation or activation. Recent evidence has suggested the involvement of the Notch pathway in IL6 signaling. The pluripotent roles of IL-6 in macrophage functions (recruitment, infiltration, phagocytosis, etc.) have been established [23]. Tumor-associated macrophages (TAMs) constitute a plastic and heterogeneous cell population of the TME that can occupy up to 50% of some solid neoplasms [37]. The Notch pathway exhibits crosstalk with the Wnt signaling cascade [38] and interferes with TME regulation and cancer stem cell maintenance [39]. The presence of a small Notch2^HIGH^ cell population in primary and bone metastatic breast cancers has been confirmed in human samples, with survival benefits for Notch2^HIGH^versus Notch2^LOW^ patients. Notch2^HIGH^ cells maintained the stem cell phenotype [18].

However, there are some limitations to the present study. First, apoptosis is related to a variety of signaling pathways. However, the relationship between the Notch signaling pathway or other pathways and apoptosis needs to be fully clarified. Furthermore, our data do not prove that *NOTCH2* P2113S is differentially expressed in different stages and histological types of human OC tissues, nor does it verify its ability to promote cancer progression in different OC cell lines.

In the future studies, we need to address several limitations of this study. First, *in-vitro* experiments should be performed on more OC cells. Second, *in-vivo* experiments were needed to confirm the roles of *NOTCH2* P2113S mutation and DAPT administration on OC progression. Considering the mechanisms, the alterations of NF-κB and Akt should also be tested in the *in-vivo* experiments.

## CONCLUSIONS

Conclusively, this paper identifies a high *NOTCH2* P2113S mutation rate in OC using WES, with these mutations associated with tumorigenesis by enhancing the ability of tumor cells to migrate, invade, and proliferate. The γ-secretase inhibitor DAPT blocks Notch2 and has the same tumorigenic effects of promoting tumor cell migration, invasion, and proliferation. The latent mechanisms might lie in reducing apoptosis through dysregulation of the BAX/BCL2 balance, affecting the repair of DNA damage by reducing DNA-PK. The *NOTCH2* P2113S mutation downregulated the expression of Notch2 and then blocked the transcription factor Hes1 along with increasing the expression of the immune regulator NF-κB P65, resulting in tumorigenesis (Figure 8). It is suggested that the Notch signaling pathway is activated to control OC cell fate. Overall, this study provides a reference for the development of clinical strategy in treating OC.

## MATERIALS AND METHODS

### Participants and sample collection

We obtained 86 SOC and paracancerous tissue specimens from 75 OC patients who received treatment in Gynecology and Obstetrics, Sichuan Academy of Medical Sciences and Sichuan Provincial People’s Hospital, University of Electronic Science and Technology of China. For WES, 22 (cancerous and pericarcinomatous tissues) specimens were collected at diagnosis from 11 patients. Another 64 OC histopathological sections were subjected to TDS. Additionally, 156 age-matched healthy women were used as controls, from whom peripheral blood was sampled for TDS. All procedures followed the 2013 Declaration of Helsinki.

### DNA separation and exome sequencing (WES)

From the same individual, DNA from primary OC tissue and matched normal counterpart was separated. The DNA library was then prepared with the use of the TruSeq DNA Sample Preparation Kit (Illumina, USA). In-solution exome enrichment was then performed using an Agilent SureSelectXT Human All Exon Kit V6, followed by DNA sequencing on a HiSeq2000 Sequencing System (Illumina, USA) with a SureSelectXT Reagent kit.

### Targeted deep sequencing (TDS)

For the purpose of detecting mutations in 220 specimens (64 cancerous tissues plus 156 normal blood samples), candidate gene screening was carried out with the use of Illumina c Bot Cluster Station/Illumina HiSeq. Then, library construction and exome enrichment were performed with the use of a NextEra Rapid Capture Exome Kit (Illumina, USA). FASTQ files were generated, and data quality was assessed to primarily evaluate NGS results. The obtained reads were then aligned with the human reference genome sequence (hg19).

### Determination of copy number (CN) variation

All primers and probes used in this section were supplied by Applied Biosystems (USA). Approximately 250 ng of DNA/specimen was hybridized using a CytoScan 750K Array (Affymetrix, USA) following relevant guidance. Data analysis with Picard (URL: https://broadinstitute.github.io/picard/) was performed, followed by normalization with the SNP-FASST2 segmentation algorithm. Visualization of the probe intensity and allele ratio was performed using Nexus *v*7.5.

### Cell cultivation and transfection

The culture medium of OC A2780 and SKOV3 cells, supplied by BeNa Culture Collection, Beijing, China, was complete Roswell Park Memorial Institute (RPMI)-1640. Plasmids used for transfection were all ordered from YouBio (Changsha, China). Then, with the use of a Q5 Site-Directed Mutagenesis Kit supplied by New England Biolabs, USA, the *NOTCH2* gene carrying p.P2113S (120459008: G>A) mutation was cloned into the pcDNA3.1 plasmids. The following primers were used: ACTACCCTTGGCATCCTTTGCCTCCTTGGCAAGGTTAGGGAGGCTAGTAG (sense) and CATGGTACTCTTGGCACTGGGCCGTCTAGACTTCTTGCCCATTGGGGTGT (anti-sense) for P2113S. After confirmation of the resulting constructs by Sanger sequencing, they were transfected into SKOV3 and A2780 cells following Lipofectamine 2000 (Invitrogen, USA) recommendations for analysis, with wild-type (WT) *NOTCH2* and empty vector plasmids as controls. The NF-κB inhibitor Bay11-7082 was purchased from MedChemExpress (Cat.NO. SF4139, USA) and dealt with the OC cells at a dose of 10 nM.

### Quantitative RT-PCR

The TRIzol reagent (Invitrogen, USA) was used for extracting total RNA the two OC cells. All procedures were conducted in line with the manufacturer’s instructions. Using the PrimeScript™ RT reagent kit (Takara, Dalian, China), the total RNA was reversely transcribed into cDNA. Next, amplification was achieved applying the SYBR Premix Ex Taq™ II (Takara) in the ABI 7500 system (Applied Biosystems, USA). The reaction conditions were as follows: 5 min at 94° C, 40 cycles of 94° C for 30 min, 55° C for 30 min and 72° C for 60 min. The products received an extension at 72° C for 10 min. A temperature of 4° C was used for storing the final products. The Primers of IL-6 was 5′- AGTCCTGATCCAGTTCCTGC-3′ and 5′-CTACATTTGCCGAAGAGCCC-3′. The internal control β-actin primers were 5′-TGGCACCCAGCACAATGAA-3′ and 5′-CTAAGTCATAGTCCGCCTAGAAGCA-3′.

### Western blotting (WB)

A2780 and SKOV3 cells transfected 48 h later were collected and lysed by RIPA (Beyotime Biotech, China). The lysates were then subjected to fractionation for extracting total protein of the whole cells and nuclear proteins. The proteins were then transferred to PVDF membranes. After being blocked in 5% non-fat milk, the membranes received cultivation (4° C) with primary antibodies targeting Notch2 (1:200; rabbit polyclonal to Notch2: ab8926, Abcam, Cambridge), DNA-PK (1:500; ab32566, Abcam, Cambridge), Hes1 (1:500; ab108937, Abcam, Cambridge), p-Akt (1:500; ab8933, Abcam, Cambridge), Akt (1:1000; ab8805, Abcam, Cambridge), Bcl-2 (1:500; ab182858, Abcam, Cambridge), Bax (1:500; ab32503, Abcam, Cambridge), NF-κB p65 (1:500; ab32536, Abcam, Cambridge), Lamin B (1:1000; ab32535, Abcam, Cambridge) and β-actin (rabbit polyclonal to β-actin: AC026, ABclonal, USA). After washing by Tris-buffered saline containing 0.1% Tween-20 (TBS-T), the membranes got 1 h of probing with the second antibody goat anti-rabbit IgG H&L (HRP; ab6721, Abcam, Cambridge) at 25° C. Subsequent membrane development were performed using the ECL kit (Cat.No.P0018S, Beyotime Biotech, China). Fusion FX7 Spectra (Vilber, France) were used to determine immunoreactivity.

### Cell proliferation (cell counting kit-8 [CCK-8]) assay

In brief, cells (3×103 per well) were seeded in triplicate onto the wells of a 96-well plate and then transfected with the P2113S mutation-bearing plasmids, WT or the empty vector. After 24, 48, 72 and 96 h, CCK-8 reagent (Cat.No. AR1160, Boster Biotech., USA) was added (110 μL/well) for cultivation (37° C) for 2 h. A Model 680 Microplate Reader (Bio-Rad, USA) was used to determine the optical density at 450 nm.

### Cell migration (scratch wound healing) assay

Cells plated in 6-well plates were grown at 2×10*^5^* cells per well, and a cell-free zone was created by scratching the cell monolayer with a 10-μL pipette tip. They were immersed in culture medium (serum-free) for incubation. An OLYMPUS IX71 inverted microscope, purchased from Olympus, Japan, was utilized to observe cell migration to the cell-free zone 24 h later, and ImageJ software (ImageJ, NIH, Bethesda, USA) was used to count the number of cells mitigated to the wounds. The experiments were repeated three times.

### Cell invasion (transwell) assay

Following Matrigel (Corning, USA) coating, 6×10^4^ transfected cells for 48 h were treated with serum-free medium suspension and added to the upper chambers. The cells attached to the upper chambers 18 h later were wiped away gently, while the transmembrane cells were treated with methanol immobilization and crystal violet (0.1%) staining. Under an inverted microscope, each specimen was observed in 10 high-power fields (×400) selected at random for analysis. The experiments were repeated three times.

### EdU labeling

For EdU labeling, 1× 10^5^ cells/well were seeded in a six-well plate. EdU (10−8 mol/L, Invitrogen, USA) was placed into the medium 24 h post transfection. The cells were fixed by 4% paraformaldehyde. After Hoechst staining, cells were then mounted in standard mounting media. The examination and photographing of the stained cells were performed with a Nikon Eclipse E600 fluorescence microscope and a Retiga 1300 Q-imaging camera, respectively. EdU-positive cell percentage = green fluorescent cell count/blue fluorescent (Hoechst stained) cell count. The experiments were repeated three times.

### DAPT and cell experiments

The DAPT powder was formulated into a solution with a final concentration of 20 μM, and DMSO was used as the solvent. DAPT powder was dissolved in 500 μL of DMSO. After complete mixing, the samples were filtered with a 0.22 μm filter membrane to remove bacteria and stored in 200 μL aliquots at -80° C for later use.

### Statistical processing

SPSS 22.0 (SPSS Inc., Chicago, USA) software was utilized for data processing. The results are presented as the mean ± standard error of the mean (SEM). All cell experiments were repeated three times, and comparisons in western blot, wound healing, CCK-8, Transwell, and EdU assays were made by Student’s t test, with statistical significance determined at P < 0.05.

### Data availability statement

The labeled dataset used to support the findings of this study is available from the corresponding author upon request.
